# Improved survival in metastatic breast cancer: results from a 20-year study involving 1033 women treated at a single comprehensive cancer center

**DOI:** 10.1007/s00432-020-03184-z

**Published:** 2020-03-18

**Authors:** Anja Welt, Simon Bogner, Marina Arendt, Josef Kossow, Antonia Huffziger, Christian Pohlkamp, Heike Steiniger, Ute Becker, Ferras Alashkar, Marzena Kohl, Marcel Wiesweg, Heike Richly, Jörg Hense, Max E. Scheulen, Martin Schuler, Siegfried Seeber, Mitra Tewes

**Affiliations:** 1 Department of Medical Oncology, West German Cancer Center, University Hospital Essen, University Duisburg-Essen, Hufelandstrasse 55, 45147 Essen, Germany; 2 Institute for Medical Informatics, Biometry and Epidemiology, University Hospital Essen, University Duisburg-Essen, Essen, Germany; 3 German Cancer Consortium (DKTK), Partner Site University Hospital Essen, Essen, Germany; 4 Department of Computer Science, University of Applied Sciences and Arts, Dortmund, Germany; 5 Department of Surgery, Herz-Jesu-Krankenhaus, Münster, Germany; 6 Department of Medicine, Augusta Krankenhaus, Düsseldorf, Germany; 7 Munich Leukemia Laboratory (MLL), München, Germany; 8 Practice for Internal Medicine, Hematology and Oncology, Oberhausen, Germany; 9 Department of Medicine I, St. Bernhard Hospital, Kamp-Lintfort, Germany; 10 Department of Hematology, West German Cancer Center, University Hospital Essen, University Duisburg-Essen, Essen, Germany; 11 Practice for General Medicine, Essen, Germany; 12 Practice for Internal Medicine, Preventicum, Essen, Germany

**Keywords:** Long-term survival, Metastatic breast cancer, New drugs

## Abstract

**Purpose:**

Diagnosis and treatment of breast cancer have changed profoundly over the past 25 years. The outcome improved dramatically and was well quantified for early stage breast cancer (EBC). However, progress in the treatment of metastatic disease has been less convincingly demonstrated. We have studied survival data of patients with metastatic breast cancer (MBC) from a large academic cancer center over a period of 20 years.

**Methods:**

Data from 1033 consecutive MBC patients who were treated at the Department of Medical Oncology of the West German Cancer Center from January 1990 to December 2009 were retrospectively analyzed for overall survival (OS) and risk factors. Patients were grouped in 5-year cohorts, and survival parameters of each cohort were compared before and after adjustment for risk factors.

**Results:**

Overall survival of patients with MBC treated at specialized center has significantly improved from 1990 to 2010 (hazard ratio 0.7, 95%CI 0.58–0.84). The increments in OS have become less profound over time (median OS 1990–1994: **24.2** months, 1995–1999: **29.6** months, 2000–2004: **36.5** months, 2005–2009: **37.8** months).

**Conclusion:**

Survival of patients with MBC has improved between 1990 and 2004, but less from 2005 to 2009. Either this suggests an unnoticed shift in the patient population, or a lesser impact of therapeutic innovations introduced in the most recent period.

## Introduction

Improved surgical, radiation and systemic therapies clearly have a positive impact on OS of women with EBC (EBCTCG 2015). However, it is still a matter of debate if survival of patients with MBC has equally improved. Since the 1990s, many new agents were introduced in the clinical care of MBC. These include taxanes, aromatase inhibitors, fulvestrant, capecitabin, vinorelbin, trastuzumab, liposomal doxorubicin, carboplatin, bevacizumab and lapatinib. Supportive therapy was optimized by recombinant granulopoiesis and erythropoiesis stimulating growth factors, and the morbidity of bone metastases was reduced by the use of bisphosphonates and denosumab. Recently, eribulin, everolimus, pertuzumab, trastuzumab emtansine (T-DM1), palbociclib, ribociclib and abemaciclib as well as atezolizumab, further expanded treatment options. This impressive therapeutic armamentarium heavily impinges on the OS outcome of randomized clinical trials conducted with new agents or combinations in earlier line treatment of MBC, as multiple options for postprogression therapy are available.

Until 2018 only very few drugs were able to demonstrate an OS benefit in clinical trials of MBC: exemestane was superior to megestrol acetate, docetaxel was more active than mitomycin/vinblastine and docetaxel plus capecitabin were more effective than docetaxel alone. In HER2-positive MBC, chemotherapy plus trastuzumab led to better survival than chemotherapy alone. Docetaxel/trastuzumab was further improved by the addition of pertuzumab and T-DM1 showed better overall survival compared to physician’s choice treatment in heavily pretreated patients (Kaufmann et al. 2000; Nabholtz et al. 1999; O’Shaughnessy et al. 2002; Slamon et al. 2001; Baselga et al. 2012; Verma et al. 2012). Still, some of these few positive studies were criticized because of clearly suboptimal second and third line treatments following progression on study therapy (O’Shaughnessy et al. 2002; Feher et al. 2005).

Recently, a significant survival benefit for a new agent could be demonstrated in MBC: Patients with hormone receptor positive tumors had improved survival with endocrine therapy (ET) plus a CDK4/6 inhibitor compared to ET alone (Im et al. 2019; Slamon et al. 2019; Sledge et al. 2019), and nab-Paclitaxel (nab-P) plus Atezolizumab was shown to be superior to nab-P alone in a defined subgroup of patients with triple negative MBC (TNBC) (Schmid et al. 2018).

Most phase III studies leading to approval of new MBC drugs have relied on superiority in surrogate endpoints such as progression-free survival (PFS). Examples include bevacizumab, fulvestrant, aromatase inhibitors, everolimus, eribulin and CDK4/6 inhibitors. Assumable, this is explained by a dilution of the effect of the respective study drug on OS because of multiple effective options for next-line therapy.

Registries of comprehensive cancer centers (CCCs) may provide a useful resource for the analysis of trends of MBC mortality over extended periods, which correlate with the availability of new therapeutic options. While patient populations treated at CCCs may be positively selected, the high level of expertise and specialization of physicians and nurses, as well as the rapid access to diagnostic and therapeutic innovations assures high quality of care. Hence, relative changes in long-term outcomes that are obtained by a stable interdisciplinary team of MBC experts are well suited to detect the survival impact of therapeutic innovations that are introduced on specific periods. Examples for such analyses have been published from several major CCCs focusing on patients with secondary (Giordano et al. 2004; Chia et al. 2007; Sundquist et al. 2017) or de novo (Dawood et al. 2015; Ruiterkamp et al. 2011; Andre et al. 2004) MBC. These studies have demonstrated a stepwise improvement of OS over several decades. However, additional studies failed to demonstrate OS improvements for the entire population of MBC patients (Nakano et al. 2015; Tevaarwerk et al. 2013) or even suggested a trend for the deterioration of OS (Ufen et al. 2014; Hölzel et al. 2017).

Against this background, we conducted a long-term analysis of survival outcomes of women with MBC treated by the breast cancer team at the Department of Medical Oncology of the West German Cancer Center from 1990 to 2009.

## Patients and methods

### Patient population

The West German Cancer Center at University Hospital Essen is one of 13 Oncology Centers of Excellence designated by the Deutsche Krebshilfe (German Cancer Aid). Routine clinical data extracted from patient charts and the electronic hospital information system of 1033 consecutive MBC patients treated at the Department of Medical Oncology from 1990 to 2009 were retrospectively analysed for probability of overall survival (OS), 4-year survival (4YS) and 5-year survival rates (5YS). This population was grouped into four cohorts based on the year of first diagnosis of metastasis (cohort 1: 1990–1994, cohort 2: 1995–1999, cohort 3: 2000–2004 and cohort 4: 2005–2009). All data analyses were conducted in a pseudonymized way. The relevant ethics committee of the Medical Faculty of the University of Duisburg-Essen (No. 17-7608-BO) approved the study.

Demographic data of patients were recorded including hormone receptor (HR) status, date of first diagnosis of breast cancer and of metastatic disease, localization of metastasis, and date of last contact or death. Particular attention was paid to high-risk patients with HR negative disease and/or visceral metastasis. HER2 status was mostly unknown in patients presenting in the years 1990–1999; accordingly, we decided to exclude HER2 status from this primary analysis.

The primary objective of the study was to evaluate the association between the period of diagnosis of MBC and survival.

### Statistics

Statistics were calculated using IBM SPSS Statistics version 21.0 (Chicago Illinois, USA) and analyzed using SAS 9.4 (SAS Institute, Cary, North Carolina). Frequencies and percentages, means, medians and corresponding standard deviations were computed as well as 4-year, 5-year and median OS whenever applicable. Kaplan–Meier survival analysis was applied to estimate cumulative probabilities of OS. The log-rank test was used to compare survival rates with respect to date of initial diagnosis, diagnosis of MBC, site of distant relapse, disease-free interval and hormone receptor status. Cox proportional hazard models were used to investigate the impact of year of diagnosis on all-cause mortality for the time intervals 1990–1994, 1995–1999, 2000–2004, and 2005–2009. As time to event, we used time to death beginning with the date of the first diagnosis of metastasis in months. We adjusted for site of metastasis, HR status and disease-free interval (DFI). *P*-values < 0.05 were considered statistically significant.

## Results

### Cohort characteristics

In total, fully evaluable data could be retrieved for 1033 consecutive patients who were all included in this analysis. Survival data were reported as of April 2016. There were 933 deaths (90.3%). The median follow-up period for surviving patients after the first diagnosis of MBC was 2.53 years. Cohort sizes were 279, 408, 143 and 203 patients for cohorts 1, 2, 3 and 4. Patient characteristics of the entire population and the four cohorts are shown in Table 1 with *p*-values from a *Z *test (percentage proportions) or *T *test (absolute numbers) of each cohort against the whole population.

**Table 1:** Characteristics of patients with metastatic breast cancer, stratified by time period of diagnosis of metastatic disease

Characteristic	Years of recurrence: No of patients (%) [*p* value for test cohort against population]				
	1990–2009 All	1990–1994 Cohort 1	1995–1999 Cohort 2	2000–2004 Cohort 3	2005–2009 Cohort 4
*N* (%)	1033 (100%)	279 (27%)	408 (40%)	143 (13.8%)	203 (19.7%)
FUP, median [months]	30.3	24.2	29.6	36.5	37.8
Age at dissemination [years]					
Median	52.2	52 [*p* = 0.55]	**50.7 [*p*< 0.01]**	**56.2 [*p*< 0.01]**	**55.1 [*p*< 0.01]**
Range	45.2–60	44.8–59.9	43.6–58	47.9–62.2	48–63.1
< 50	421 (40.8%)	114 (40.9%) [*p* = 0.97]	**193 (47.3%) [*p*= 0.03]**	49 (34.3%) [*p* = 0.11]	**65 (32%) [*p*= 0.02]**
≥ 50	612 (59.2%)	165 (59.1%)	215 (52.7%)	94 (65.7%)	138 (68%)
Hormone receptor *N* (%)					
Positive	613 (59.3%)	148 (53.1%) [*p* = 0.07]	229 (56.1%) [*p* = 0.30]	**101 (70.6%) [*p*= 0.01]**	**135 (66.5%) [*p* = 0.03]**
Negative	307 (29.7%)	70 (25.1%) [*p* = 0.10]	136 (33.3%) [*p* = 0.27]	34 (23.8%) [*p* = 0.13]	67 (33%) [*p* = 0.40]
Unknown	113 (10.9%)	**61 (21.9%) [*p*< 0.01]**	43 (10.5%) [*p* = 0.82]	**8 (5.6%) [ *p*= 0.04]**	**1 (1%) [ *p*< 0.01]**
First metastasis *N* (%)					
Metastasis as disease recurrence	907 (87.8%)	246 (88.2%)	368 (90.2%)	120 (83.9%)	173 (85.2%)
Metastasis at initial diagnosis of breast cancer	126 (12.2%)	33 (11.8%) [*p* = 0.89]	40 (9.8%) [*p* = 0.20]	23 (16.1%) [*p* = 0.20]	30 (14.8%) [*p* = 0.31]
DFI* *N* (%)					
< 18 months	210 (20.3%)	70 (25.1%) [*p* = 0.08]	96 (23.5%) [*p* = 0.18]	**17 (11.9%) [*p*= 0.02]**	**27 (13.3%) [p = 0.02]**
18–35 months	226 (21.9%)	66 (23.7%) [*p* = 0.47]	99 (24.3%) [*p* = 0.35]	26 (18.2%) [*p* = 0.31]	35 (17.2%) [*p* = 0 0.13]
≥ 36 months	471 (45.6%)	110 (39.4%) [*p* = 0.06]	173 (42.4%) [*p* = 0.27]	77 (53.9%) [*p* = 0.07]	**111 (54.7%) [*p* = 0.02]**
DFI, median [months]	30.9	**25.9 [*p*< 0.01]**	**27.6 [*p*< 0.01]**	**38 [*p*< 0.01]**	**41 [*p*< 0.01]**
Localization of first metastasis					
Bone only	215 (20.8%)	68 (24.4%) [*p* = 0.28]	76 (18.6%) [*p* = 0.67]	24 (16.8%) [*p* = 0.27]	47 (23.2%) [*p* = 0.52]
Liver and/or lung	608 (58.9%)	152 (54.5%) [*p* = 0.13]	261 (64%) [*p* = 0.08]	83 (58%) [*p* = 0.81]	112 (55.2%) [*p* = 0.29]
Other	210 (20.3%)	59 (21.1%) [*p* = 0.71]	71 (17.4%) [*p* = 0.19]	36 (25.1%) [*p* = 0.16]	44 (21.7%) [*p* = 0.51]

Significant deviations of cohort characteristics versus the whole population are high-lighted in bold

*DFI* interval from first diagnosis of breast cancer to first diagnosis of metastasis, *FUP* interval from diagnosis of metastasis to last contact or death

Patient characteristics were not evenly distributed across the four cohorts. The median age at the time of diagnosis of metastatic disease ranged from 52 to 56 years with significant differences. The proportion of patients aged < 50 years, representing mostly patients with premenopausal status, decreased over time from cohort 1 (114 pts, 40.9%, *p* = 0.97), cohort 2 (193 pts, 47.3%, *p* = 0.03), cohort 3 (49, 34.7%, *p* = 0.11) to cohort 4 (65, 32%, *p* = 0.02).

There was a clear time trend in the proportion of patients with unknown HR status: In the first cohort (1990–1994), nearly 22% of patients had unknown HR status. This fraction steadily decreased to < 1% in the most recent cohort (2005–2009). Consequently, the proportion of patients with HR positive disease was higher in cohorts 3 (71%, *p* = 0.01) and 4 (67%, *p* = 0.03) as compared to cohorts 1 (53%) and 2 (56%).

The fraction of patients with HR negative disease has a larger measurement error due to its smaller weight. Comparison of cohort 4 to cohort 1, however, shows a proportional increase compatible with the hypothesis of stable underlying characteristics subject to more widespread testing.

The proportion of patients within each cohort who presented with MBC at initial diagnosis ranged from 9.8% (cohort 2) and 11.8% (cohort 1) to 16.1% (cohort 3) and 14.8% (cohort 4).

Testing the cohorts against the entire population reveals non-significant p-values well above 5% (Table 3). A test of the largest difference, cohort 3 against cohort 2, gives the marginal *p*-value of 0.053.

For the complementary large fraction of patients with metastatic recurrence, the DFI was detailed for each cohort and showed a significant shift from early (DFI < 18 months) to late recurrence (DFI > 36 months). The more recent cohorts thus have a significant (*p* < 0.01) longer median DFI which increased from 25.9 to 27.6, 38 and 41 months in the cohorts 1, 2, 3 and 4, respectively.

No statistically significant differences were observed between the four cohorts with respect to the site of metastatic disease at the time of the first diagnosis of metastasis. Visceral metastasis was present in 54% to 64% of patients with no relevant change over time. “Bone only” metastasis were seen in 17% to 24% of patients without a relevant change over time.

### Survival estimates and risk factors

Overall survival from the first diagnosis of metastatic disease was compared between the four cohorts (Table 2). The median OS was 24.2 months, 29.6 months, 36.5 month and 37.8 months in patients who developed metastasis during 1990–1994, 1995–1999, 2000–2004 and 2005–2009, i.e. in cohorts 1, 2, 3 and 4. Overall survival differed significantly between the four cohorts (*p* < 0.0001). 4YS rates were 22.6%, 29.7%, 36.4% and 37.4%, 5YS rates were 14%, 23%, 30.8% and 24.6%, respectively (see Table 2). The site of metastasis, HR status and DFI were identified as significant risk factors for OS (Table 2, bottom).

**Table 2:** Unadjusted outcome and risk factors

Group of patients	*N*	OS, median [months]	4YS [%]	5YS [%]	Logrank *p*-value
Total	1033	30.3	30.4	22	
1990–1994	279	24.2	22.6	14	< 0.0001
1995–1999	408	29.6	29.7	23	
2000–2004	143	36.5	36.4	30.8	
2005–2009	203	37.8	37.4	24.6	
Bone only	215	44.8	48.8	36.7	< 0.0001
Liver and/or lung	608	24.7	23.5	16.5	
Other	210	31.8	29.1	21	
HR +	613	37.3	36.4	25.9	< 0.0001
HR −	307	19.7	14.3	10.1	
HR unknown	113	31.1	37.2	29.2	
Initial metastasis	126	38.6	38.1	29.4	< 0.0001
DFI < 18 month	210	18.4	17.1	11.4	
DFI 18–35 month	226	25.4	21.7	15	
DFI > 35 month	471	37.5	37.4	27.2	
Age < 50 years	421	29.6	28.7	20.9	0.6087
Age ≥ 50 years	612	31.6	30.7	22.1	

Median overall survival, probability of 4-year and 5-year overall survival of patients with metastatic breast cancer, according to time period of diagnosis of metastatic disease and according to prognostic factors

*OS* overall survival. 4YS, 4-year overall survival. *5YS* 5-year overall survival

**Fig. 1:**
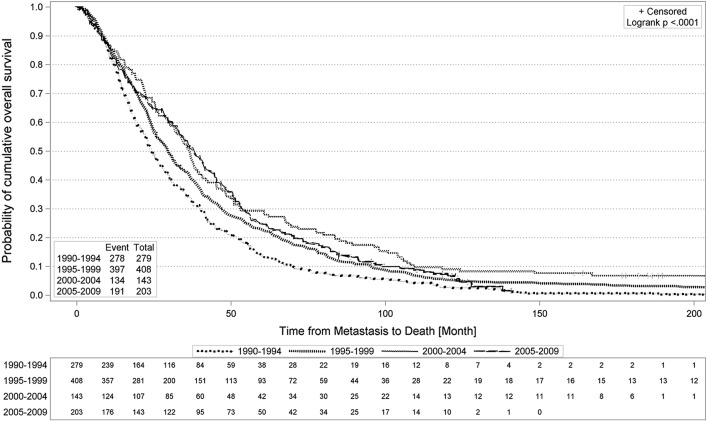
Probability of overall survival of patients with metastatic breast cancer stratified by time period of diagnosis of metastatic disease using Kaplan–Meier Analysis

Kaplan–Meier survival curves for OS for the four cohorts are shown in Fig. 1. Survival for cohort 2 was above that of cohort 1, as was survival of cohort 3 relative to cohort 2. The surviving fraction of the most recent cohort 4 hardly differs from its previous cohort 3. The corresponding curves cross multiple times.

Table 3 shows the cohort-to-cohort comparison of OS. Data are given both for unadjusted survival (top) as well as after adjustment (bottom) for the risk factors “site of metastasis”, “HR status” and “DFI”, as identified in Table 2. Risk adjustment for relevant variables shall account for the differences in the distribution of risk factors between cohorts as shown in Table 2 and described above.

**Table 3:** Overall survival comparison cohort-to-cohort over time, before and after risk adjustment

cohort	1990–1994	1995–1999	2000–2004	2005–2009
Incremental change in OS over time, hazard ratios, unadjusted; HR given for column compared to row (< 1: better);				
1990–1994	1 (Reference)	**0.78 (0.66; 0.90)**	0.63 (0.51; 0.77)	0.70 (0.58; 0.84)
1995–1999	1.29 (1.11; 1.51)	1 (Reference)	**0.81 (0.67; 0.99)**	0.90 (0.76; 1.07)
2000–2004	1.59 (1.29; 1.96)	1.23 (1.01; 1.50)	1 (Reference)	**1.11 (0.89; 1.39)**
2005–2009	1.43 (1.19; 1.73)	1.11 (0.94; 1.32)	0.90 (0.72; 1.13)	1 (Reference)
Incremental change in OS over time, hazard ratios adjusted for risk factors “site of metastasis”, “HR status” and “DFI until metastasis”; HR ratios given for column compared to row (< 1: better)				
1990–1994	1 (Reference)	**0.71 (0.61; 0.84)**	0.66 (0.53; 0.82)	0.70 (0.58; 0.85)
1995–1999	1.40 (1.20; 1.64)	1 (Reference)	**0.92 (0.76; 1.18)**	0.99 (0.83; 1.18)
2000–2004	1.52 (1.23; 1.88)	1.08 (0.89; 1.33)	1 (Reference)	**1.07 (0.86; 1.34)**
2005–2009	1.53 (1.27; 1.85)	1.06 (0.98; 1.27)	0.91 (0.73; 1.13)	1 (Reference)

Most important results in incremental change in OS over time are high-lighted in bold

In the unadjusted analysis, we find a significant increase of OS from cohort 1 to cohort 2 (HR 0.78 [0.66; 0.90]) and again, slightly smaller, from cohort 2 to cohort 3 (HR 0.81 [0.67; 0.99]). Cohort 4, by contrast, does not differ from cohort 3 (HR 1.1 [0.89; 1.39]). After risk-adjustment, this picture remains intact with a further shift of the weight of gain in OS towards earlier years (cohort 1 to cohort 2: HR 0.71, significant; cohort 2 to 3: HR 0.92, overlapping with unity; cohort 3 to 4: HR 1.07, overlapping with unity).

Comparison of the most recent cohort 4 to the most remote cohort 1 shows a stable and significant long-term improvement in OS (unadjusted HR 0.70 [0.58; 0.84]); risk-adjusted HR 0.70 [0.58; 0.85]).

## Discussion

In the current data set, we have observed an effect of year of diagnosis of MBC on OS. This is in line with findings from other high volume and expertise centers demonstrating a modest but significant increase in survival over time among patients with MBC. Giordano et al. published a seminal study of patients treated at the MD Anderson Cancer Center. Survival of 834 patients with recurrent breast cancer improved from 1974 to 2000 with 1% reduction in the risk of death for each successive year (2003). A study group from British Columbia found a relative prolongation of approximately 30% in OS for 2.150 patients with MBC diagnosed between 1991 and 2001 (Chia et al. 2007). A Swedish study group demonstrated improved survival from 13 to 33 months and 5YS from 10 to 27% for 784 patients diagnosed with MBC from 1985 to 2014 (Sundquist et al. 2017). However, not all of the prior analyses were adjusted for DFI and other covariates. Several major CCCs focused on patients with de novo MBC also reporting an improvement of OS for the entire population (Ruiterkamp et al. 2011; Dawood et al. 2015; Andre et al. 2004).

There is evidence that the most striking effect is given in the HER2 positive subset (Tevaarwerk et al. 2013; Nakano et al. 2015; Sundquist et al. 2017), partly represented as “HR negative disease” because information regarding HER2 status was missing in some analyses (Tevaarwerk et al. 2013). Clearly, this subgroup of patients has shown an OS benefit obviously caused by the introduction of HER2-targeted therapies (Slamon et al. 2001; Dawood et al. 2010; Baselga et al. 2012).

In general, more patients were treated with systemic therapies in the neo-/adjuvant setting since the 1990s. This is consistent with the international trend of decline in breast cancer mortality in western countries (DeSantis et al. 2014; Carioli et al. 2017) which cannot only be contributed to the implementation of mammographic screening in the more recent years. On the other hand, the extensive use of systemic treatment in the neo-/adjuvant setting in recent years could have reduced the effectiveness of palliative systemic therapy in these groups, because a greater proportion of patients were no longer naïve for chemotherapy and endocrine treatment. Ufen et al. studied 1635 patients treated for MBC in three German cancer centers between 1980 and 2009. They failed to find an improvement but observed a decline of median OS over time which was associated with a shift towards more aggressive disease (2014). This corresponds with Hölzel et al. reporting a clear improvement of 5-year-survival in EBC over three decades, presumably caused by better perioperative systemic treatment leading to an extension of DFI. Paradoxically, this effect was accompanied by a reduction of median OS in patients with MBC (2017).

We here present 1033 consecutive patients with MBC treated between 1990 and 2009 at a major German comprehensive cancer center. No predefined sequence of treatment for metastatic breast cancer was applied. Treatment sequence was individualized as a function of prior treatment, tumor biology, the dynamics of metastasis, and patient preferences (particularly in relation to toxicities and treatment intervals) by a stable expert team.

Cohort 2 (1995–1999) was much larger than other cohorts. During this period our cancer center offered high-dose chemotherapy with autologous stem cell transplantation in clinical studies as part of the therapeutic concept (Bojko et al. 2004). This is probably the main reason why more patients were assigned to our clinic.

Patient characteristics were unequally distributed across the four cohorts regarding HR status, age and DFI. The growing proportion of patients with known HR positivity may rather be explained by the adoption of routine testing of HR expression than by a real change in the distribution of HR positivity over time.

The significant increase in age over time may partially reflect the longer DFI between diagnosis and treatment of the primary breast tumor and metastasis. This is consistent with well-recognized improvements of multimodal treatment of primary breast cancer. More generally, this trend of rising age may indicate that the increase in life expectancy at birth for the overall population is not lost on women with MBC.

We find significant and relevant improvement in OS from 24.2 months to 38.8 months over this period of two decades. Our analyses with respect to the time of presentation (4 cohorts of distinct 5-year periods) and established risk factors (age, HR status, DFI, site of metastasis) showed that the gain in OS slowed over time. After risk adjustment, accounting for fluctuations over time in the patient characteristics, a significant gain is shown for the years 1995–1999 when compared to 1990–1995, and a numerical, statistically non-significant but plausible gain for the period of 2000–2004 compared to 1995–1999. No further improvement is seen when cohort 4 (2005–2009) is compared to the immediately preceding cohort. However, the improvement in OS accrued in the first 15 years of the study sustains.

In agreement with Hölzel et al. (2017) our analyses showed a sustained increase of DFI over time from 25.9 (1990–1994) to 41.0 months (2005–2009), plausibly due to improvements in the primary treatment. Assuming the recurrent population is composed of more negatively selected cancer biologies, this could explain that increments in OS have become less profound over time despite of the introduction of new therapeutic agents in the treatment of MBC in Germany (1990–1994: paclitaxel; 1995–1999: docetaxel, letrozole, anastrozole; 2000–2004: exemestane, capecitabin, trastuzumab, fulvestrant, pegylated liposomal doxorubicin, gemcitabin; 2005–2009: bevacizumab, liposomal doxorubicin, lapatinib, nab-P).

We recognize that the current study has limitations. Some degree of bias may be attributed to the retrospective character of this analysis. Additionally, HER2 status was not included in our evaluation, because it was unknown in many patients treated for MBC in the nineties. Therefore a subsequent analysis regarding HER2 status of patients in cohorts 3 and 4 is planned soon. This will include a more recent cohort (2010–2014), too.

The strengths of this study include a large unselected patient population and extensive median follow-up time (median years: 2.53; range 1.30–4.49). OS results of the cohorts 3 (2000–2004; OS 36.5 months) and 4 (2005–2009; OS 37.8 months) are well in line with those reported from the prospective German TMK cohort study, namely median OS of 33.8 months reporting 1395 “real world” patients treated from 2007 to 2015 in various oncology practices and community hospitals in Germany (Fietz et al. 2017).

In sum, we report survival data of patients with MBC treated at the Department of Medical Oncology at the West German Cancer Center that show large improvements between 1990 and 2009. During this time, multiple effective anticancer agents that can palliate MBC have been introduced into routine practice. The rate of improvement, however, appears to have been slowing during the observation time of our study. This suggests either a negative selection of patients with recurrent breast cancer due to more effective perioperative therapy over time in EBC or a lesser impact of therapeutic innovations introduced in the most recent period. Since 2010, again many drugs were released in the treatment of MBC (eribulin, T-DM1, pertuzumab, everolimus, ribociclib, palbociclib, abemaciclib, atezolizumab) which hopefully will further improve survival with MBC.
